# LVA for Advanced Unilateral Lower Extremity Lymphedema: Impact of ICG Lymphography of Normal Side in Improving the Lymphatic Detection Rate and Operative Time

**DOI:** 10.1055/a-2511-8588

**Published:** 2025-04-01

**Authors:** Usama Abdelfattah, Tarek Elbanoby, Mona Omarah, Saber M. Abdelmaksoud, Eatmad Allam, Serag Monir

**Affiliations:** 1 Department of Plastic and Reconstructive Surgery, Al-Azhar University, Cairo, Egypt; 2 Department of Plastic and Reconstructive Surgery, El Nile Insurance Hospital, Cairo, Egypt; 3 Department of Plastic and Reconstructive Surgery, Port Said University, Port Said, Egypt; 4 Department of Physiotherapy, Al-Azhar University, Cairo, Egypt

**Keywords:** LVA, advanced lymphedema, lower extremity

## Abstract

**Background** Indocyanine green (ICG) lymphography has limited use in the detection of functioning lymphatics in advanced lymphedema. This study presents the use of normal-side ICG lymphography to navigate the potential sites of functional lymphatics and reports its impact on the lymphatic detection rate and operative time.

**Methods** This was a retrospective study of unilateral lower extremity late-stage II or III lymphedema patients who underwent lymphaticovenous anastomosis (LVA) between February 2018 and June 2022.

Markings for possible lymphatic vessels were made on the affected side solely in the early group (2018–2019) and on both the affected and normal side in the late group (2020–2022) using ICG lymphography.

**Results** Between 2018 and 2022, 86 patients had complete data for analysis. Dermal backflow stage III was present in 5 limbs (5.81%), stage IV in 40 limbs (46.51%), and stage V in 41 limbs (47.67%). The late group had a higher mean lymphatic detection rate, which was statistically significant in the proximal leg incision site (2.05 ± 0.91 vs. 0.74 ± 0.82; *p* = 0.041). There was a significant tendency toward lower total LVA operative time per limb in the late group, which was led by the normal side mapping, with a mean operative time of 158 ± 14.88 minutes compared with 199 ± 12.45 minutes in the early group ( *p* = 0.035).

**Conclusion** Mirroring the affected limb by utilizing the normal-side ICG lymphography in guiding the incision sites for LVA could improve the lymphatic detection rate, minimize the number of incisions, and shorten the operative time.

## Introduction

Lymphedema is a chronic, gradually debilitating condition caused by primary or secondary lymphatic drainage system dysfunction. The International Society of Lymphology (ISL) staging scale (0–3) is currently used to diagnose and guide lymphedema management. 1 Lymphaticovenous anastomosis (LVA) is a procedure that is known to be beneficial mostly for patients with early-stage lymphedema. 2 3 In patients with advanced lymphedema, it is hard to find functional lymphatics candidates for effective LVA; many surgeons perform vascularized lymph node transfer (VLNT) or debulking surgeries instead of LVA. 4 5 6 However, there are several limitations to VLNT, such as reported variable outcomes, probable donor site morbidity, poor cosmetic appearance, and a lack of unanimity over the recipient site. 7 8 9 Liposuction and debulking are two other options for advanced lymphedema treatment. Nevertheless, these operations are not physiological; they require long-term compression garments, which are difficult for most patients in our region's somewhat humid weather and leave unattractive scars, as in the Charles procedure. Recently, there has been a paradigm shift, and LVA has been shown to be successful in advanced-stage extremity lymphedema. 10 11 This concept arose from enhanced imaging studies that combined duplex ultrasound, magnetic resonance lymphangiography, and indocyanine green (ICG) lymphography to identify deep functional lymphatic candidates for effective LVA. 12 13 14 15 The purpose of this retrospective study was to present the clinical, intraoperative findings, and operative time of LVA in patients with advanced-stage unilateral lower extremity lymphedema (LEL) who underwent normal-side ICG lymphography.

## Methods

Patients with unilateral LEL who were refractory to compression therapy, classified as late stage II or III by the ISL, and underwent LVA between February 2018 and June 2022 were reviewed retrospectively after receiving approval from the Institutional Review Board at Al-Azhar University (2022-1181). Clinical data, ICG lymphographic staging, intraoperative data, and limb volumes were collected. Exclusion criteria included (1) early-stage lymphedema or a linear pattern only on ICG lymphography, (2) bilateral LEL, and (3) prior LVA or other lymphedema surgeries. All patients provided written consent for the operation, use of data, and their lower extremities' photos.

### Preoperative Preparation

**Video 1** ICG lymphography of the affected limb (right side) showing a stardust pattern of dermal backflow (stage IV), with no linear pattern observed. ICG, indocyanine green.


**Video 2** ICG lymphography of the normal lower limb (left side) of the same patient showing the normal linear pattern of lymphatic flow. ICG, indocyanine green.


**Fig. 1:**
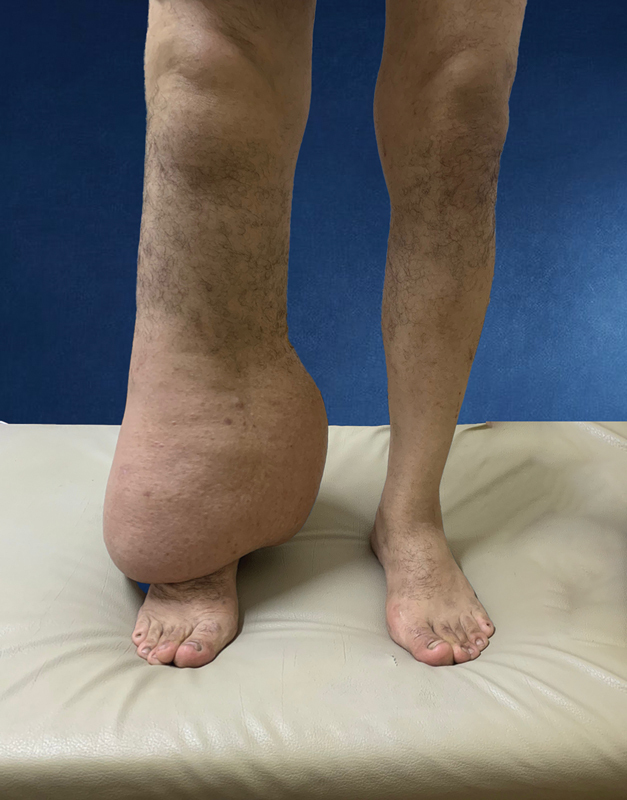
A male patient (52 years) with skin change, minimal pitting, and advanced fibrosis is shown with late-stage II lymphedema of the right leg. The patient's condition progressed gradually to its current state over 9 years.

**Fig. 2:**
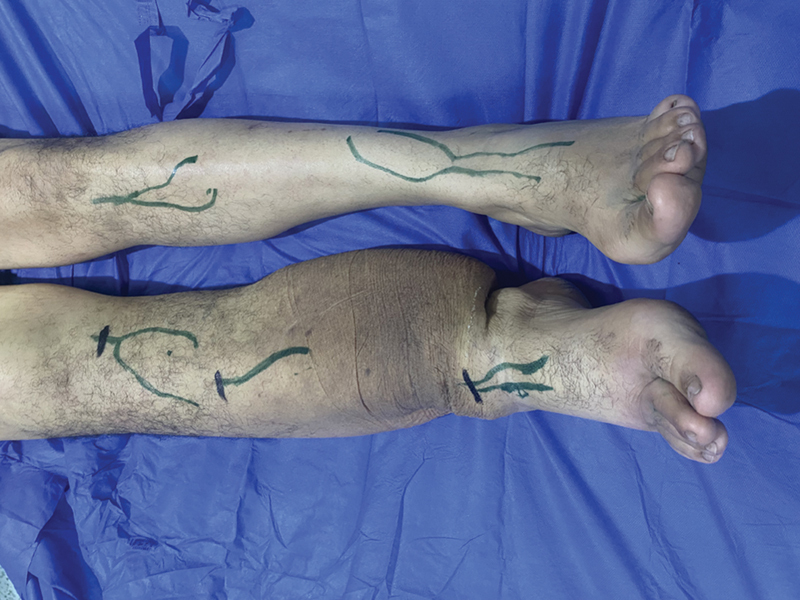
Marking of the affected limb after mirroring the normal side with determination of incision sites based on the mirroring.

Before surgery, all patients underwent lymphoscintigraphy. ICG lymphography was also performed the night before surgery: 0.2 ml of ICG (PULSION Medical Systems, Feldkirchen, Germany) was injected subdermally at the first and third web spaces of the foot and the lateral border of the Achilles tendon into the affected limb only in the early group (2018–2019). In the late group (2020–2022), ICG was injected into the bilateral lower extremities in a similar way as early group. A near-infrared camera system was used to take fluorescence images of lymph flows circumferentially (Fluobeam, Fluoptics, Grenoble, France). Images of ICG lymphography were taken early and at a plateau phase (12 hours after injection). The ICG lymphographic findings on the affected side were used to define the dermal backflow stage. Markings for possible lymphatic vessels were made on the affected side solely in the early group and on the normal side in the late group using ICG lymphography ( Figs. 1 and 2 and Videos 1 and 2 ).

### Intraoperative Approach

The lymphaticovenular anastomosis (LVA) incision site was determined as the most proximal site of any linear pattern observed. In the early group, the locations of LVA were determined along the long saphenous vein and Seki point 16 in cases of dermal backflow with no linear lymphatics observed. However, starting in February 2020, the normal side was used as a reference and mirrored its marking on the affected side as a guide in determining the incision site for LVA. In this late group, we chose incision sites at dermal backflow regions with linear lymphatics on the normal side. A few minutes before surgery, 0.1 to 0.2 ml of a solution of 2.5% of isosulfan blue dye (Blue Patent V Sodique, Guerbet, Le Raincy, France) was injected into the web spaces, and a few centimeters distal to planned LVA incision sites, of the affected limbs at the subdermal and subcutaneous levels. This solution is often absorbed by functioning lymphatics, making it easier to distinguish functioning lymphatics from nonfunctioning sclerotic lymphatic channels. Functional lymphatics that are reliable for LVA were often blue-colored visualized upon a skin incision, and lymphatic fluid flows out when opened under the microscope; in this circumstance, the lymphatic detection was defined as “affirmative.”

### Data Collection

In the clinical and intraoperative data, the age, body mass index (BMI), duration of lymphedema, ISL stage, history of cellulitis, anatomical sites of the incision, ICG lymphography pattern at the incision site, and finding of a lymphatic vessel were all reviewed. The operating times were estimated by reviewing the medical records to figure out how long the whole surgery took. We calculated the operative time from the skin incision to closure due to the nature of how surgical times were recorded in the operative record, including anesthesia time, preparation time, the aggregate time required for LVA, confirmation of patency, and closure. The LEL index was determined preoperatively and then postoperatively at 3 months, 6 months, and 1 year, and for the study, the reduction in volume was compared between the two groups. The unpaired student's *t* -test, Mann–Whitney U test, or Fisher's exact test were all used for statistical analysis, as appropriate. All of the tests were two-sided, with the significance level set at 0.05. The statistical software tool SPSS, version 25, was used for all analyses (IBM Corporation, Chicago, IL).

## Results

Between 2018 and 2022, 92 cases received LVA for late-stage II or III unilateral lymphedema, with 86 having complete operative data accessible for study. The average age and BMI were 42.4 years and 29.9 kg/m ^2^ , respectively ( Table 1 ). Dermal backflow stage III was seen in 5 limbs (5.81%), stage IV in 40 limbs (46.51%), and stage V in 41 limbs (47.67%). A bivariate analysis was used to examine the demographic characteristics of two groups: the early (control) and the late. There were no statistically significant differences between the two groups in terms of age, BMI, lymphedema duration, or degree of dermal backflow ( Table 2 ). LVA was attempted through 3.31 ± 0.45 mean incision sites per limb in the early group, with a mean blue lymphatic detection of 3.52 ± 1.42, while in the late group, which was guided by the normal side, the mean incision sites was 2.27 ± 0.24 with a greater overall mean lymphatic detection of 4.82 ± 1.78. The late group had a higher mean lymphatic detection rate, which was statistically significant in the proximal leg incision site (2.05 ± 0.91 vs. 0.74 ± 0.82; *p* = 0.041; Table 3 ). There was a significant tendency toward lower total LVA operative time per limb in the late group, which was led by the normal side, with a mean duration of 158 ± 14.88 minutes compared with 199 ± 12.45 minutes in the early group ( *p* = 0.035; Table 3 ). Overall, in the late group guided by the normal side mapping, the total number of functional blue lymphatic vessels detected and LVA performed per leg increased, but overall operational time and the number of incision sites decreased. Postoperative outcome changes in lymphedema severity are shown for each patient group in Table 4 . Affected limb circumference was reduced at 3 months in all 49 patients in the late (mirrored) group compared with 89.2% in the early group ( *p* = 0.0762), while by 12 months lymphedema had recurred in only 1 patient compared with 3 patients with lymphedema recurrence in the early group ( *p* = 0.0866). The mean reduction in the LEL index was 6.42 ± 4.38 ( *n* = 37) versus 8.42 ± 4.26 ( *n* = 49; *p* = 0.018) at 3 months, and 8.68 ± 6.22 ( *n* = 37) versus 10.23 ± 6.16 ( *n* = 49; *p* = 0.0634) at 1 year. Cellulitis developed only in one patient in the early group 7 months after surgery.

**Table 1:** Patient demographics

	2018–2019	2020–2022	*p* -value
Total number	37	49	0.156
Age (mean ± SD, years)	42 ± 8.5	43 ± 11.2	0.223
BMI (mean ± SD, years)	29 ± 4.6	31 ± 5.2	0.198
Sex			
Male	15	21	0.404
Female	22	28	0.267
Affected side			
Right	20	22	0.374
Left	17	27	0.396
Type			
Primary	9	15	0.277
Secondary	28	34	0.402
Mean duration of lymphedema ± SD, years	8.2 ± 5.4	7.6 ± 6.6	0.389
Cellulitis	32 (86.5%)	41 (83.7%)	0.605
Leg dermal backflow stage			
0	0	0	–
I	0	0	–
II	0	0	–
III	2	3	0.542
IV	18	22	0.176
V	17	24	0.112

Abbreviations: BMI, body mass index; SD, standard deviation.

**Table 2:** Percentage of each International Society of Lymphology stage with dermal backflow stage III to V

Group A				Group B		
	Leg dermal backflow stage			Leg dermal backflow stage		
	III	IV	V	III	IV	V
ISL						
IIb III	2 (100%) 0	16 (88.9%) 2	3 (17.6%) 14 (82.4%)	3 (100%) 0	4 (18.2%) 18 (81.8%)	2 (8.3%) 22 (91.7%)

Abbreviation: ISL, International Society of Lymphology.

**Table 3:** Surgical details of lymphaticovenous anastomosis

	Early group (2018–2019)	Late group (2020–2022)	*p* -value
Incision site			
Total	3.31 ± 0.45	2.27 ± 0.24	0.104
Foot/ankle	1.02 ± 0.14	1.0 ± 0.00	0.654
Proximal leg	1.15 ± 0.46	1.03 ± 0.32	0.820
Groin	1.16 ± 0.55	0.24 ± 0.2	0.135
Lymphatic detection rate (mean)			
Total	3.52 ± 1.42	4.82 ± 1.78	0.062
Foot/ankle	2.11 ± 1.6	2.38 ± 2.1	0.92
Proximal leg	0.74 ± 0.82	2.05 ± 0.91	0.041 *
Groin	0.67 ± 0.35	0.41 ± 0.44	0.604
Number of LVAs performed (mean)			
Total	3.44 ± 1.22	4.36 ± 1.88	0.142
Foot/ankle	2.1 ± 1.72	2.02 ± 2.1	0.535
Proximal leg	0.68 ± 0.92	1.82 ± 0.72	0.041 *
Groin	0.66 ± 0.24	0.52 ± 0.41	0.852
Lymphatic vessel diameter, mm	0.63 ± 0.28	0.69 ± 0.36	0.322
Mean overall operative time	199 ± 12.45	158 ± 14.88	0.035 *

Abbreviation: LVA, lymphaticovenous anastomosis.

* indicating statistical significance.

**Table 4:** Postoperative outcome in the early group and late (mirrored) group

	Early group	Late (mirroring) group	*p* -value
3 months after surgery	*n* = 37	*n* = 49	
Limb volume reduction	33/37 (89.2%)	49/49 (100%)	0.0762
Change in LEL index	6.42 ± 4.38 (−6.28 to 11.18)	8.42 ± 4.26 (4.22 to 15.62)	0.0746
Cellulitis	None	None	
6 months after surgery	*n* = 37	*n* = 49	
Limb volume reduction	35/37 (94.6%)	48/49 (97.9%)	0.152
Change in LEL index	5.48 ± 9.39 (−11.36 to 10.18)	9.44 ± 3.22 (−2.36 to 12.66)	0.0458 ^a^
Cellulitis	None	None	
1 year after surgery	*n* = 37	*n* = 49	
Limb volume reduction	34/37 (91.9%)	48/49 (97.9%)	0.0866
Change in LEL index	8.68 ± 6.22 (−9.42 to 14.56)	10.23 ± 6.16 (−1.54 to 26.32)	0.0634
Cellulitis	1 attack	None	

Abbreviation: LEL, lower extremity lymphedema.

## Discussion

VLNT has traditionally been used to treat moderate to severe lymphedema (late stage II or III, ISL); however, the results have been mixed, with a paucity of reports of long-term functional outcomes. 17 18 19 LVA was not typically performed on patients with advanced-stage lymphedema due to increased subcutaneous fibrofatty deposits and pathological alterations in the superficial lymphatics that made identifying functional lymphatics difficult. Recently, breakthroughs in microscopic magnification and imaging technologies have led to the spread of supermicrosurgery, and LVA has proven to be beneficial in advanced stage II and III extremity lymphedema. 10 11 17

Many factors, like the BMI, the number of cellulitis attacks, and the degree of dermal backflow, are strongly related to the detection rate of functioning lymphatics that are candidates for LVA. 20 In the early stages of lymphedema, ICG lymphography is likely to show a linear pattern with less thickness of the fibrofatty deposition, so the lymphatic vessel detection rate is high. 20 21 Due to the limited penetration effect of ICG lymphography, the thickness of subcutaneous fat increases in higher BMI and advanced lymphedema patients (dermal backflow stages III–V), making it more challenging for a surgeon to detect functional lymphatic channels. The difficulty increased when seeking deep ones beneath the superficial fascia, which is more effective for LVA in advanced lymphedema. 10 22 There are few studies in the literature that investigate the factors that influence the lymphatic detection rate. However, Yamamoto et al published a multivariate analysis and reported an inverse relation between the lymphatic detection rate and higher BMI and advanced degrees of dermal backflow. 20 According to our results, using the normal-side ICG lymphography as a guide in speculation of potential functioning lymphatic sites on the affected side resulted in a higher detection rate of functional lymphatic vessels (4.82 ± 1.78 vs. 3.52 ± 1.42) with a significant improvement in the detection rate at the proximal leg region (2.05 ± 0.91 vs. 0.74 ± 0.82, *p* = 0.041). The proximal leg region has the thickest subcutaneous fat with the most difficult region to identify linear lymphatics compared with the foot or groin region, which could explain the significant improvement in lymphatic identification in this region.

In this study, we found that the late group operated more efficiently in terms of a lower number of incisions, a higher rate of lymphatic identification, a higher number of LVAs per limb, and a significant reduction in operative time.

One potential argument is that the improved operative time was due to a consistent team or improved surgeon experience, but this was not the case because the circulating nurse and surgical technologist were not fixed throughout the evaluation dates and trainees were routinely involved in all cases, which has also been shown to increase operative times. 23 Few studies have investigated the parameters that influence LVA operative times. Pereira et al reported a retrospective study on the operative time and volume reduction of affected extremities, the results showed that the surgeon's experience had no impact on the results of LVA, whereas the operative time per LVA was significantly improved with increased surgeon experience. 24 Nevertheless, this study included four different surgeons, and the majority of patients had early-stage I or II lymphedema.

The lower limb lymphatic drainage pattern was defined by anatomical dissection and injection studies of the superficial lymphatics in human cadavers. The lymphatic system in the lower extremity begins distally with collector vessels running on the lateral and medial sides of each toe, then runs proximally over the dorsum and anterior portion of the ankle. Following the course of the long saphenous vein, with little running on the posterior thigh, the vessels concentrate mostly on the anteromedial and posterior regions of the leg, knee, and thigh. Finally, lymph flows to the superficial inguinal nodes. 25 Despite the extensive description of anatomical lymphatic drainage patterns in the superficial tissues of the limbs, little attention was paid to lymphatic drainage symmetry. Except for the lower anterior torso and a small area of the posterior torso, most skin areas with sufficient data revealed symmetric drainage, according to one anatomical study by Reynolds et al. 26 Dealing with the left/right symmetry of the lymphatic pathways of the upper limbs, many studies support this feature in healthy persons. 27 Gentileschi et al, reported a similar approach in 16 patients with stage IIb (ISL) upper limb lymphedema, for every limb, they were able to find 3.6 ± 0.73 incision sites each containing at least one lymphatic vessel suitable for anastomosis. 28 Regarding the lower limb, one study reported predictive lymphatic mapping in patients with advanced unilateral lower limb lymphedema. They used the mirroring approach in only five cases of unilateral lymphedema of the lower extremities. In no cases, they failed to find a lymphatic channel suitable for LVA. 29

The retrospective nature of our study limited the data that could be obtained on team dynamics, such as communication patterns and disruptions that could affect operative time. The improved surgeon and institutional experience over the study period may possibly have an impact on operation duration. However, due to the high-volume nature of our lymphedema program and the surgeon's expertise prior to the examined periods, this source of bias was deemed to be reduced. Furthermore, surgical trainees were present in all reported cases, which may have an impact on operating room communication, team interaction, and operative efficiency.

Although duplex ultrasonography is now a useful tool in lymphatic imaging and LVA planning, image quality is mostly dependent on the operator's experience, and a learning curve is needed. In addition, ultrasound-based identification of tiny lymphatic vessels is still challenging. 14 Nonetheless, ICG lymphography is still the gold standard and most widely used modality in lymphatic imaging due to its simplicity, sensitivity, and accuracy in staging and planning for LVA. Combining ICG lymphography and duplex ultrasonography would be more accurate and useful. Another limitation in our study is that the results have limited generalizability, more extensive multicenter investigations involving more surgeons are necessary.

### Conclusion

Despite the limitations of ICG lymphography in advanced lymphedema, using the normal side ICG lymphography can overcome this limitation. Mirroring the affected limb by utilizing the normal side mapping in guiding the incision sites for LVA could improve the lymphatic detection rate, minimize the number of incisions, and shorten the operative time.
